# The tf/Nrf2/GSTP1 pathway is involved in stress‐induced hepatocellular injury through ferroptosis

**DOI:** 10.1111/jcmm.18494

**Published:** 2024-06-18

**Authors:** Xiaofei Tian, Yingmin Li, Lei Lei, Xiaowei Feng, Hongjian Xin, Hao Chen, Guozhong Zhang, Min Zuo, Weibo Shi, Bin Cong

**Affiliations:** ^1^ Hebei Key Laboratory of Forensic Medicine, Collaborative Innovation Center of Forensic Medical Molecular Identification, Department of Forensic Medicine Hebei Medical University Shijiazhuang China; ^2^ Department of Forensic Medicine Hebei North University Zhangjiakou China

**Keywords:** ferroptosis, liver injury, restraint stress, TF/Nrf2/GSTP1

## Abstract

Stress triggers a comprehensive pathophysiological cascade in organisms. However, there is a substantial gap in the research regarding the effects of stress on liver function. This study aimed to investigate the impact of restraint stress on hepatocellular damage and elucidate the underlying molecular mechanisms. An effective mouse restraint stress model was successfully developed, and liver function analysis was performed using laser speckle imaging, metabolomics and serum testing. Alterations in hepatocyte morphology were assessed using haematoxylin and eosin staining and transmission electron microscopy. Oxidative stress in hepatocytes was assessed using lipid reactive oxygen species and malondialdehyde. The methylation status and expression of *GSTP1* were analysed using DNA sequencing and, real‐time PCR, and the expression levels of GPX4, TF and Nrf2 were evaluated using real‐time quantitative PCR, western blotting, and immunohistochemical staining. A stress‐induced model was established in vitro by using dexamethasone‐treated AML‐12 cells. To investigate the underlying mechanisms, GSTP1 overexpression, small interfering RNA, ferroptosis and Nrf2 inhibitors were used. GSTP1 methylation contributes to stress‐induced hepatocellular damage and dysfunction. GSTP1 is involved in ferroptosis‐mediated hepatocellular injury induced by restraint stress via the TF/Nrf2 pathway. These findings suggest that stress‐induced hepatocellular injury is associated with ferroptosis, which is regulated by TF/Nrf2/GSTP1.

## 1 INTRODUCTION

In response to physiological stress, there is a substantial increase in glucocorticoid (GC) levels.^1^ High levels of GC act on vital organs, such as the brain, heart, kidneys and liver, mobilizing energy to meet demands while also regulating inflammatory responses and cellular functions.^2^ It inhibits the activity of mitochondrial electron transport chain complexes, leading to an increase in reactive oxygen species (ROS).^3^ This induces cellular stress responses, ultimately resulting in damage to crucial organs. Intense and prolonged stress can lead to hepatocellular injury,^4^, ^5^ however, the mechanisms underlying this damage remain complex and are not fully understood. The dynamic equilibrium of iron ions plays a pivotal role in maintaining normal cellular functions. Under pathological conditions, the disruption of iron metabolism leads to the accumulation of free iron in cells.

Ferroptosis^6^, ^7^, ^8^ differs from apoptosis, necrosis and autophagy regarding morphological, biochemical, and genetic characteristics.^9^ It is linked to various oxidative stress, antioxidant defence pathway, metabolism, gene transcription, and protein degradation mechanisms. Ferroptosis is associated with elevated levels of intracellular or mitochondrial ferrous ions (Fe^2+^), lipid peroxidation with changes in long‐chain fatty acid‐CoA ligase 4 (ACSL4) expression, accumulation of lipid ROS, loss of antioxidant defence functions, and aberrant expression of ferroptosis‐related proteins.^10^, ^11^, ^12^ Transferrin (TF) is an abundant metal‐binding protein in serum.^13^ Primarily synthesized in the liver, TF binds to iron (Fe^3+^) in a soluble and non‐toxic form during circulation,^14^, ^15^ facilitating the transport of iron to the bone marrow and other tissues. Elevated levels of aspartate aminotransferase (AST) and alanine aminotransferase (ALT) were observed in a mouse model of liver injury induced by iron overload. Additionally, ROS and malondialdehyde (MDA) accumulate in hepatocytes.

Glutathione S‐transferase Pi (GSTP1), encoded by the glutathione S‐transferase (GST) Pi gene, plays a crucial role in detoxification by combating oxidative damage and the development of various diseases.^16^, ^17^ It enhances the cellular antioxidative capacity and is one of the most extensively studied members of the GST family. GSTs catalyse the conjugation of glutathione (GSH) to a broad spectrum of ROS, hydrogen peroxide, and toxic substances, thus protecting hepatocytes against oxidative stress.^18^ Hypermethylation of GSTP1 leads to a loss of function and increases susceptibility to liver cancer.^19^ Nuclear factor erythroid 2‐related factor 2 (Nrf2) is a critical regulator of oxidative stress that is usually exhibits low expression.^20^ When exposed to a stimulus, it is highly expressed, activates the endogenous antioxidant pathway, binds to antioxidant response elements, initiates transcription, and induces the production of a series of antioxidant enzymes, such as heme oxygenase 1 (HO‐1),^21^ superoxide dismutase 1 (SOD1), and GSTP1,^22^, ^23^ to alleviate oxidative damage. Nrf2 promotes the expression of glutathione peroxidase 4 (GPX4),^24^ enhances intracellular antioxidant capacity and mitigates cellular injury.

However, the mechanisms underlying hepatocellular injury caused by prolonged and continuous stress remain unclear. Given that the liver is a storage organ for iron ions, we hypothesized that stress could lead to the accumulation of Fe^2+^, hypermethylation, and low GSTP1 expression in hepatocytes, thereby activating the Nrf2 response to participate in hepatocyte injury. To verify this hypothesis, we evaluated changes in hepatocellular injury. We investigated the dynamic changes in GSTP1, Nrf2, and ferroptosis‐related proteins to successfully establish a restraint stress model in vivo and in vitro. These findings contribute to further clarification of the molecular mechanisms underlying hepatocellular injury.

## 2 MATERIALS AND METHODS

### 2.1 Animal models

Male C57BL/6 mice (8 weeks of age; body weight 20–22 g) were purchased from Beijing Vital River Laboratory Animal Technology Co., Ltd. (Beijing, China) and housed under standard laboratory conditions with a 12‐h light/12‐h dark cycle at constant temperature and humidity. Food and water were provided ad libitum. The mice were randomly allocated into six groups: the control group, groups exposed to continuous stress for 24 h (24h), groups confined continuously for 6 h per day for 7 days (7d) or 21 days (21d), and respective control groups (*n* = 10/group). A cylindrical acrylic glass tube with a length of 10 cm and inner diameter of 2.5 cm was used for the restraint stress. The tube body was provided with air holes and movable valves at both ends of the tube so that the mice could drill into the tube and could not move or turn freely. To further investigate the role of ferroptosis in stress injury, two additional groups of mice were included as follows: the 24 h + Fer‐1 group (24 h + Fer‐1) and the 24 h + DMSO group (24 h + DMSO) (*n* = 10/group). Fer‐1 is Ferrostatin‐1, a specific inhibitor ferroptosis. The solution was dissolved in DMSO. All procedures were performed in accordance with National Institutes of Health guidelines and approved by the Hebei Medical University Institutional Review Board for Animal Experiments (IACUC‐Hebmu‐2023011).

### 2.2 Liver blood flow (LBF) determination

LBF was measured at baseline using a laser Doppler perfusion imager (PeriCam PSI NR; PSIN‐01104, Sweden) equipped with a computer. The scanner head was positioned 15 cm from the liver. The beam illuminated the tissues to a depth of 0.5 mm. The magnitude of liver blood flow was represented by different colours, with blue and red denoting low and high blood flow, respectively. A rectangle region (1.5 × 1.5 cm, the centre of the left lateral lobe) including the main branch of the microcirculatory network was outlined on each image and used to calculate the area‐averaged flux. Data were presented as mean flux from the measured region in perfusion units.^25^

### 2.3 Biochemical analysis

Fe^2+^, MDA, and total GSH (T‐GSH)/GSSG in the liver were assayed using assay kits (A039‐2, A003‐1, and A061‐1, Jiancheng Bioengineering Institute, China), according to the manufacturer's recommended protocol. Serum ALT and AST levels were determined using commercial enzymatic kits (Rayto, China) and an automatic biochemical analyser (Chemray 240).

### 2.4 Histopathological analysis

Paraffin‐embedded liver tissue sections were sliced at a thickness of 4.5 μm and stained with haematoxylin and eosin to visualize the morphology of the cells in the tissue. Livers were fixed with 2.5% glutaraldehyde in 0.1 mol/L of Sorenson's buffer (0.1 mol/L H_2_PO_4_, 0.1 mol/L HPO_4_ [pH 7.2]) for 4 h, washed 3× with PBS and then treated with 1% OsO_4_ in 0.1 mol/L Sorenson's buffer for 2 h at 25°C. After dehydration using an ethanol series and acetone (100%), the livers were embedded in acetone and embed‐812 (SPI). Thin sections were obtained using an ultramicrotome (Leica), and stained with 2% uranyl acetate and lead citrate. Images were obtained using a Hitachi transmission electron microscopy (TEM).

### 2.5 Metabonomics

The mice were anaesthetised and sampled, and their livers were immediately removed and frozen in liquid nitrogen. Metabolomics was performed using the Shanghai Zhong Ke New life. All differentially expressed metabolites were subjected to heat map and KEGG ontology enrichment analyses. For KEGG enrichment analysis, *p* < 0.05 was used as the threshold to determine statistically significant enrichment of the gene sets.

### 2.6 Cell culture, viability assay, and transfection

AML12 (alpha mouse liver 12) cells, hepatocytes isolated from the normal livers of 3‐month‐old mice, were purchased from the ATCC and cultured in DMEM/F‐12 (Gibco) supplemented with 10% fetal bovine serum (Gibco) and 50 μg/mL of penicillin/streptomycin and 1% Insulin‐Transferrin‐Selenium Supplement 100X and 40 ng/mL dexamethasone (DEX). DEX was used to induce AML‐12, and the optimal concentration and time of DEX administration were determined using the CCK8 assay. To knock down or overexpress target proteins, Lipofectamine 3000 (Thermo Fisher Scientific, L3000008), small interfering RNA (siRNA), and tagging plasmids (pcDNA‐flag‐GSTP1) were used to transfect AML‐12 cells. The groups included control, DEX, DEX+Fer‐1, DEX+ML‐385, DEX+Fer‐1+ML‐385, overexpression GSTP1 (flag‐GSTP1), DEX+GSTP1 overexpression (flag‐GSTP1+DEX), knock down GSTP1 expression with siRNA (siRNA GSTP1), and the control group of siRNA (siRNA NC).

### 2.7 Real‐time polymerase chain reaction

Total RNA was extracted from the mouse livers using TRIzol (Invitrogen), and reverse transcribed using TransScript One‐Step gDNA Removal (Takara). The cDNAs were used for PCR with TB Green® Premix Ex Taq™ (Takara). Expression of the target gene was mainly calculated using 2^−ΔΔct^ normalization of the glyceraldehyde 3‐phosphate dehydrogenase (*GAPDH*) gene. The primer pair sequences used for RT‐PCR are listed in Table 1.

**TABLE 1:** RT‐PCR primers of TF, GPX4, ACSL4, GSTP1, and GAPDH.

Name	sequence	length
GPX4‐F	5′′‐TGCAACAGCTCCGAGTTCCT‐3′	143
GPX4‐R	5′‐GTGACGATGCACACGAAACC‐3′	
TRF‐F	5′‐ACATCCCTATGGGCATGCTG‐3′	123
TRF‐R	5′‐GTGGGCCAATACACAGGTCA‐3′	
ACSL‐F	5′‐ACCAACCCCTTCAGACATGG‐3′	114
ACSL‐R	5′‐TCACACTGGCCTGTCATTCC‐3′	
GSTP1‐F	5′‐CCTCTGTCTACGCAGCACTGAATC‐3′	100
GSTP1‐R	5′‐AGCATTCGCATGGCCTCAC‐3′	
GAPDH‐F	5′‐AAATGGTGAAGGTCGGTGTGAAC‐3′	90
GAPDH‐R	5′‐CAACAATCTCCACTTTGCCACTG‐3′	

### 2.8 Immunohistochemistry

The isolated mice liver was fixed in 10% formalin, and the tissue was subsequently dehydrated in steps of ethanol and embedded in paraffin. The liver wax block was sectioned into continuous sections (5 μm) for immunohistochemistry. The sections were deparaffinized with xylene and alcohol and incubated in 0.01 mol/L citrate buffer (pH 6.0) for microwave antigen repair. The liver slices were incubated in an endogenous peroxidase blocker solution for 30 min. Next, non‐specific binding sites were blocked with normal goat serum for 40 min at 37°C, and incubated at 4°C overnight with the primary antibodies: rabbit anti‐GPX4 (Proteintech, 30388‐1‐AP, China; 1:200), mouse anti‐TF (Proteintech, 17435‐1‐AP; 1:200), mouse anti‐ACSL4 (Proteintech, 22401‐1‐AP; 1:100). The following day, after PBS washing the slices incubated with the corresponding secondary antibodies: HRP‐labelled goat anti‐rabbit IgG and HRP‐labelled goat anti‐mouse IgG for 40 min at 37°C. The slices were then washed with PBS and DAB for 3–5 min. The wells were then rinsed with tap water, counterstained, dehydrated, made transparent, and sealed with coverslips.

### 2.9 Western blotting

Mouse liver tissues were mechanically homogenized with 10× tissue lysis RIPA buffer (Beyotime, P0013B, China) containing phenylmethanesulfonyl fluoride (Beyotime, ST506). After being placed on the ice for 30 min, the lysates were centrifuged for 5 min at 14000 × g and 4°C. The supernatants were then collected and cryopreserved at −80°C until used. Protein concentrations were calculated using a BCA protein assay kit (Beyotime, P0010S). Equal amounts of total protein were loaded onto 6%–15% polyacrylamide gels. The proteins were separated using SDS–PAGE and transferred by electrophoresis to polyvinylidene fluoride membranes (Bio Rad, 1620177, USA), and blocked with 5% non‐fat milk at 37°C for 1 h. The membranes were incubated with anti‐β actin (Proteintech, HRP‐60008; 1:1000), anti‐GPX4 (1:500), anti‐TF (1:1000), anti‐ACSL4 (1:2000), anti‐Nrf2 (Proteintech, 80,593‐1‐PBS, China; 1:1000), anti‐HO‐1 (Proteintech, 10701‐1‐AP; 1:2000), anti‐GSTP1 (Proteintech, 15902‐1‐AP; 1:1000) overnight at 4°C. The following day, the membranes were incubated with the HRP‐labelled secondary antibodies for 1 h at 37°C, away from light. Specific protein bands were visualized using an Amersham Imager 600 (America). ImageJ software (NIH) was used to quantify and analyse the protein bands (NIH).

### 2.10 Fluorescence staining

Liver sections and AML‐12 cells were fixed with 4% paraformaldehyde for 10 min and washing in PBS, slides were incubated with C11‐BODIPY (Invitrogen™ D3861, 5 μmol/L; Cayman No.27086, 30 mg/mL) for 30 min at 37°C. After washing with PBS, DAPI was used to stain the cell nuclei. Images were obtained using an Olympus BX61VS Microscope or a Leica TCS SP8 Microscope. ImageJ software was used for quantification and fluorescence signal analysis.

### 2.11 Detection of *GSTP1* methylation and its expression at mRNA level

The primer pairs used to detect *GSTP1* methylation are listed in Table 2. Genomic DNA was extracted, treated with disulfite, amplified, and cloned into the pSWE‐Topo Zero Vector. Preparation and transformation of the receptive state, PCR identification of the bacterial solution, and sequence determination.

**TABLE 2:** The primer pair sequences used for GSTP1 methylation detection.

Name	Sequence	Length
M‐GSTP1‐BSP‐F	5′‐TTAGTATAAAGTGGAAGGGAGTTGG‐3′	391
M‐GSTP1‐BSP‐R	5′‐CCCCAAACTCCTATTACAAACTACC‐3′	

### 2.12 Statistical analysis

All data were shown as the mean ± SEM and analysed by SPSS 25.0 (IBM SPSS Statistics, Chicago, IL, USA). GraphPad Prism (version 8.0; GraphPad Software Inc., CA, USA) was used for graph generation. Statistical comparisons were performed using one‐way or two‐way ANOVA, followed by Tukey's post hoc test for multiple comparisons. Differences were considered statistically significant at *p* < 0.05.

## 3 RESULTS

### 3.1 GSTP1 methylation contributed to stress‐induced hepatocellular damage and dysfunction

**FIGURE 1:**
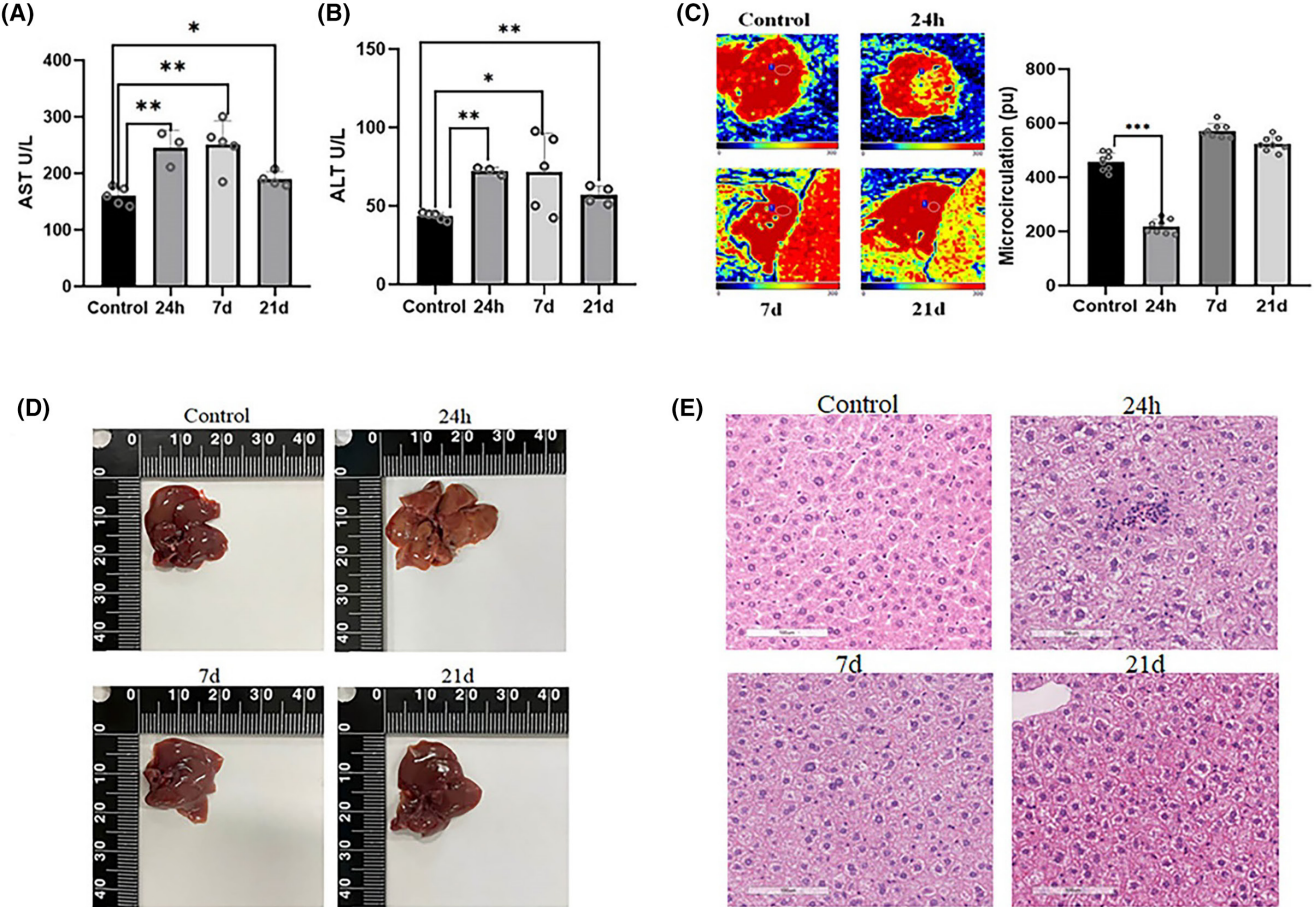
Stress resulted in hepatocyte injury. (A) and (B) Serum levels of ALT and AST were significantly increased by restraint stress in mice (*n* = 4). (C) Liver blood flow (LBF) was measured using a Laser‐Doppler Perfusion Imager (*n* = 8). (D) Gross visual observation of the liver. (E) HE staining of hepatocytes. Scale bar = 100 μm. The values are expressed as the mean ± SEM, *p < 0.05, **p < 0.01, ***p < 0.001 versus the control group. ALT, alanine aminotransferase; AST, aspartate aminotransferase; HE, haematoxylin and eosin.

To investigate the effect of stress on hepatocytes, we first measured the serum levels of AST and ALT after different restraint stresses, which could reflect the damage to liver function. As shown in Figure 1A,B, restraint stress significantly increased the serum levels of AST (*p* < 0.05) and ALT (*p* < 0.05) in mice. Using Doppler perfusion imaging, we found that 24 h of continuous stress apparently reduced hepatic blood flow, whereas 6 h of daily stress continuously for 7 or 21 days did not significantly alter it (Figure 1C), and this was confirmed by visual observation of the liver (Figure 1D). In addition, using haematoxylin and eosin staining, we confirmed that sustained 24 h stress caused damage to hepatocytes (Figure 1E). Therefore, we focused on the molecular mechanisms underlying hepatocellular damage induced by sustained 24 h stress exposure.

**FIGURE 2:**
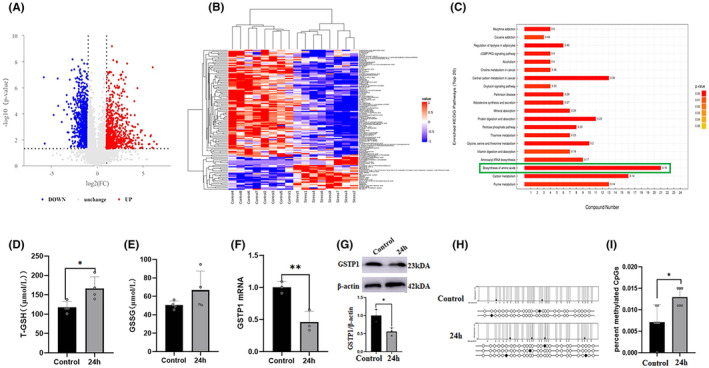
GSTP1 methylation contributed to stress‐induced hepatocyte dysfunction. (A) The volcanic map of liver metabolite differences due to restraint stress. (B) Heat maps of metabolites with significant differences in negative ion patterns. (C) The KEGG enrichment pathway was mapped according to the differences in liver metabolites between the restraint stress and normal control groups. (D) and (E) Detection of T‐GSH and GSSG in hepatic tissue from control and restraint (24 h) groups (*n* = 4). (F) and (G) The expression of GSTP1 in hepatic tissue of mice (*n* = 3). (H) and (I) The methylation of GSTP1 in mice liver (*n* = 4). The values are expressed as the mean ± SEM, *p < 0.05, **p < 0.01 versus the control group.

To better analyse the effect of stress on liver function, we performed metabolomics and found that sustained 24 h of stress significantly perturbed hepatic metabolism (Figure 2A,B), especially amino acid biosynthesis (Figure 2C). Kyoto Encyclopedia of Genes and Genomes analysis revealed that these metabolic abnormalities were closely related to GSH levels. Therefore, we detected the levels of GSH and found that stress elevated T‐GSH (*p* < 0.05), but did not significantly increase GSSG (*p* > 0.05; Figure 2D, E). GSTP1 catalyses the nucleophilic addition of glutathione sulfur thiolate to a wide range of electrophilic substrates, forming less toxic and more soluble compound.^26^ Thus, GSTP1 is a key enzyme involved in the conversion of GSH to GSSG. The stress‐induced reduction of GSTP1 was responsible for T‐GSH accumulation and the ineffective conversion of GSH to GSSG (Figure 2F,G). By further detecting the methylation level of GSTP1, we confirmed that stress‐induced elevation of GSTP1 methylation was an important cause of reduced GSTP1 enzymatic activity (Figure 2H,I).

### 3.2 Ferroptosis was involved in hepatocyte hyperoxidation‐induced liver injury in stressed mice

**FIGURE 3:**
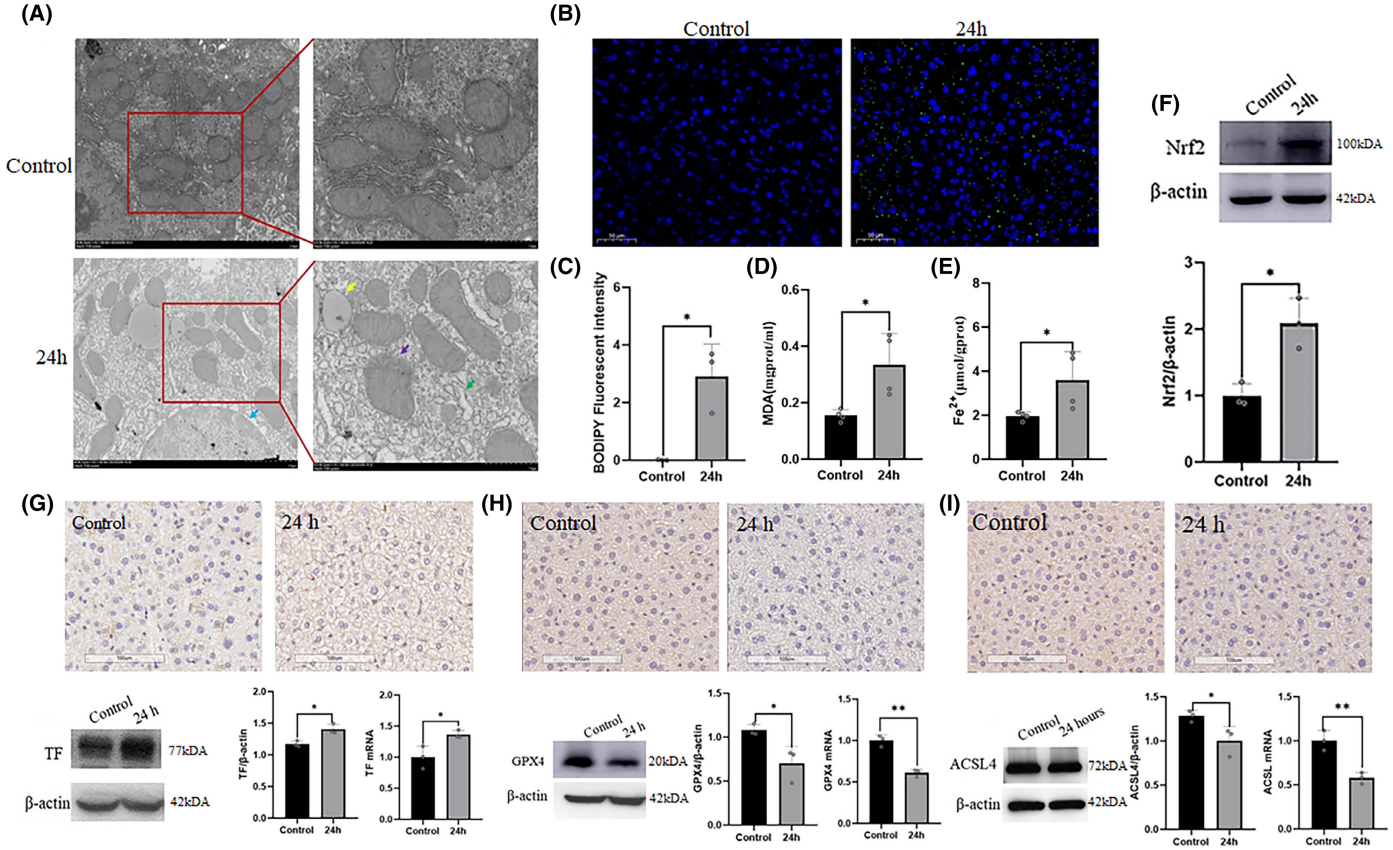
Ferroptosis was involved in hepatocyte injury induced by stress. (A) The result of TEM about the ultrastructural changes of hepatocytes in the 24 h and control groups. (B) and (C) Immunofluorescence results of lipid ROS probe C11‐bodipy, green is C11‐bodipy, blue is DAPI. Scale bar = 50 μm. (D) and (E) Detection of MDA and Fe^2+^ ions in hepatic tissue from control and restraint groups (*n* = 4). F: the expression of Nrf2 at the protein level in hepatic tissue of different groups (*n* = 3). (G–I): The expression of TF, GPX4 and ACSL4 at protein and mRNA levels in hepatic tissue from control and restraint group (*n* = 3). Scale bars = 100 or 2.0 μm. The values are expressed as the mean ± SEM, *p < 0.05, **p < 0.01 versus the control group. MDA, malondialdehyde.

GSH directly influences oxidative respiratory chain processes in the mitochondria. TEM revealed that the mitochondrial membrane was dissolved and blurred, and that lipid droplets were generated in the cytoplasm after stress exposure (Figure 3A). Stress resulted in an obvious increase in lipid ROS (*p* < 0.05) and MDA (*p* < 0.05) levels in the hepatocytes (Figure 3B–D). Furthermore, stress significantly elevated the expression of Nrf2 (*p* < 0.05; Figure 3F), which plays an antioxidant role in hepatocytes. These data suggested that stress leads to hyperoxidation of hepatocytes.

As the liver is a major repository of iron ions, we explored whether ferroptosis was involved in stress‐induced hepatocyte hyperoxidation. Consequently, we detected Fe^2+^ and ferroptosis‐related proteins in hepatocytes and observed a notable elevation in Fe^2+^ (*p* < 0.05; Figure 3E) and TF (*p* < 0.05), as well as a marked reduction in GPX4 (*p* < 0.05) and ACSL4 (*p* < 0.05; Figure 3F–I). These findings suggested that stress activates ferroptosis in hepatocytes.

**FIGURE 4:**
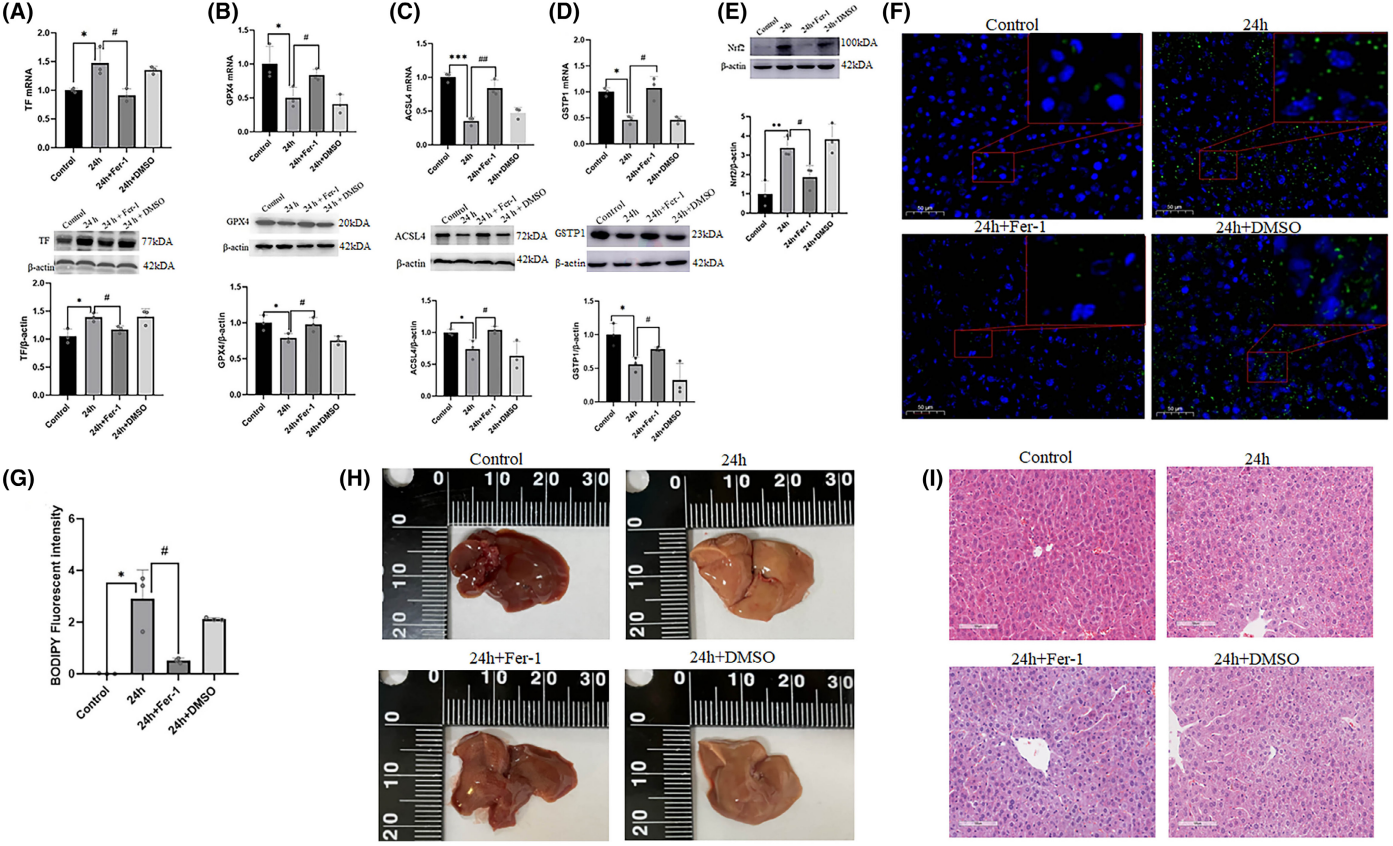
Fer‐1 markedly mitigated the stress‐induced ferroptosis. (A–E) The expression of TF, GPX4, ACSL4, GSTP1 and Nrf2 in hepatic tissue from control group, restraint stress group, Fer‐1 group and DMSO group (*n* = 3). (F) and (G) Immunofluorescence results of lipid ROS probe C11‐bodipy (green), blue is DAPI. Scale bar = 50 μm. (H) Gross morphology of hepatic tissue from control group, 24 h of restraint stress group (24 h), 24 h of restraint stress + Fer‐1 group (24 h + Fer‐1) and 24 h of restraint stress + DMSO group (24 h + DMSO). (I) The HE staining of hepatic tissue from groups of control, 24 h, 24 h + Fer‐1 and 24 h + DMSO. Scale bar = 100 μm. Values are expressed as the mean ± SEM, *p < 0.05, **p < 0.01, ***p < 0.001 versus the control group, ^#^ *p* < 0.05, ^##^ *p* < 0.01 versus the 24 h + Fer‐1 group. HE, haematoxylin and eosin.

To clarify the role of ferroptosis in hepatocyte hyperoxidation, we used Fer‐1 to inhibit ferroptosis. Fer‐1 markedly mitigated the stress‐induced increase in TF (*p* < 0.05) and the corresponding decrease in GPX4 (*p* < 0.05) and ACSL4 (*p* < 0.05; Figure 4A–C). Moreover, Fer‐1 successfully reversed the stress‐induced decrease in GSTP1 (*p* < 0.05) and Nrf2 (*p* < 0.05; Figure 4D,E). Further examination of lipid ROS levels revealed a notable reduction (*p* < 0.05) following Fer‐1 treatment (Figure 4F,G). These findings strongly suggested that Fer‐1 mitigated the hyperoxidative state of stressed hepatocytes. These observations were further supported by haematoxylin and eosin and gross visual observations, confirming the alleviation of liver injury (Figure 4H,I).

### 3.3 The TF/Nrf2/GSTP1 pathway regulated ferroptosis and participated in stress‐induced hepatocellular injury

**FIGURE 5:**
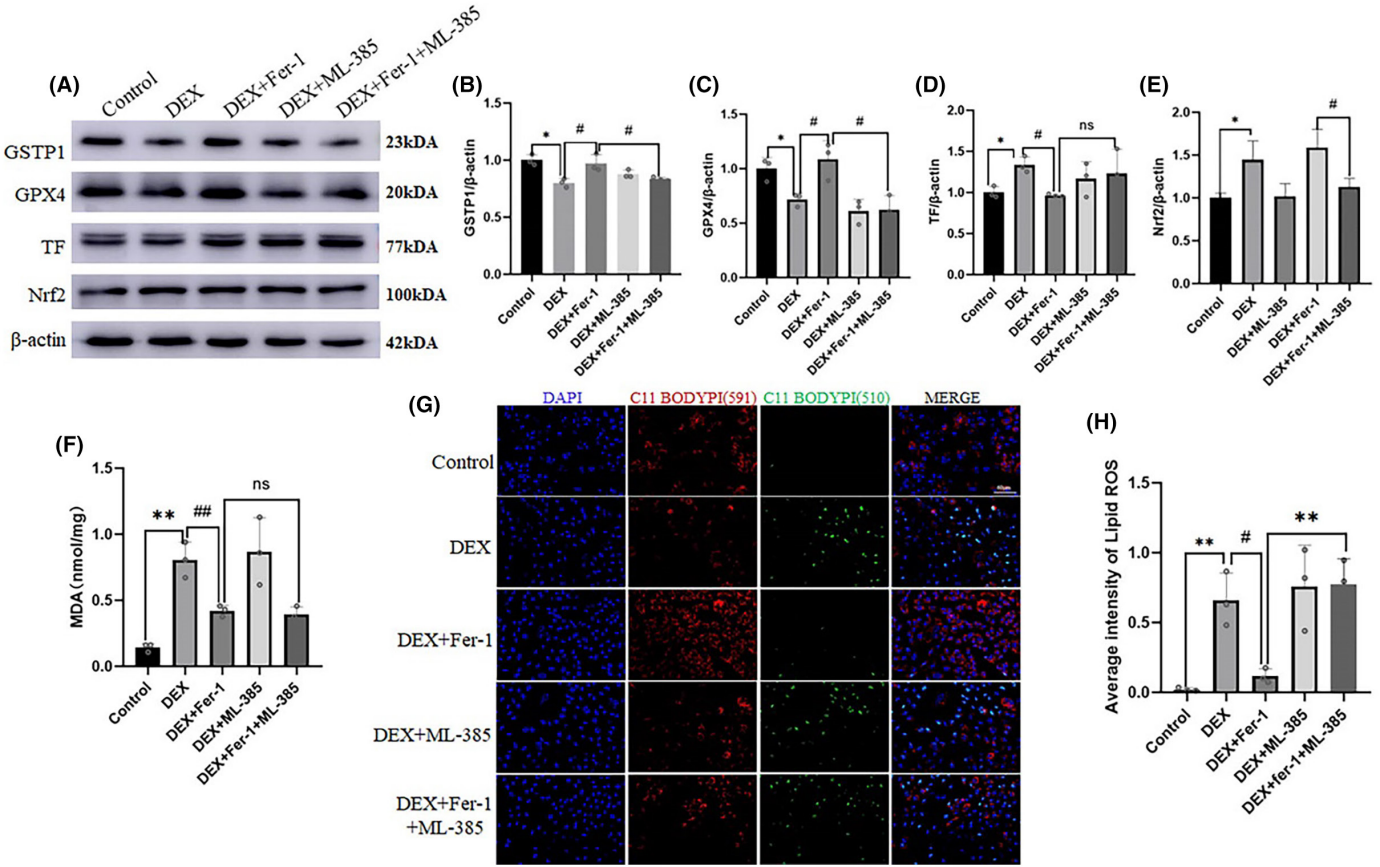
Fer‐1 alleviate the expression changes of ferroptosis‐related proteins induced by DEX, but ML‐385 weakened the remission effect of Fer‐1. (A–E) The expression of Nrf2, GPX4, GSTP1 and TF at the protein level in AML‐12 cells (*n* = 3). (F) The content of MDA in AML‐12 cells of different groups (*n* = 3). (G) and (H) The content of C11 BODIPY in AML‐12 cells of different groups. Scale bar = 60 μm. Values are expressed as the mean ± SEM, *p < 0.05, **p < 0.01 versus the control group. ^#^ *p* < 0.05, ^##^ *p* < 0.01 versus the DEX + Fer‐1 group. DEX, dexamethasone.

To further investigate the interaction among TF, Nrf2 and GSTP1, AML‐12 cells were treated with DEX to establish an in vitro stress model. DEX reduced the expression of GPX4 (*p* < 0.05) and GSTP1 (*p* < 0.05), and increased the expression of Nrf2 (*p* < 0.05) and TF (*p* < 0.05). When Fer‐1 was co‐treated with DEX on AML‐12 cells, Fer‐1 alleviated the changes in the expression of GSTP1 (*p* < 0.05), GPX4 (*p* < 0.05), and TF (*p* < 0.05) induced by DEX. To explore the interaction between Nrf2 and GSTP1, AML‐12 cells were simultaneously treated with the Nrf2 inhibitors, ML‐385, Fer‐1, and DEX. The results indicated that ML‐385 inhibited the changes in GSTP1and GPX4, which were alleviated by Fer‐1 (*p* < 0.05; Figure 5A–E). This suggested that Nrf2 regulated the expression of GSTP1. We examined the effects of DEX on MDA and lipid ROS levels in AML‐12 cells. DEX significantly increased lipid ROS (*p* < 0.05) and MDA (*p* < 0.05) levels in AML‐12 cells, while the addition of Fer‐1 alleviated lipid ROS. However, ML‐385 weakened the remission effect of Fer‐1 (*p* < 0.05; Figure 5F–H).

**FIGURE 6:**
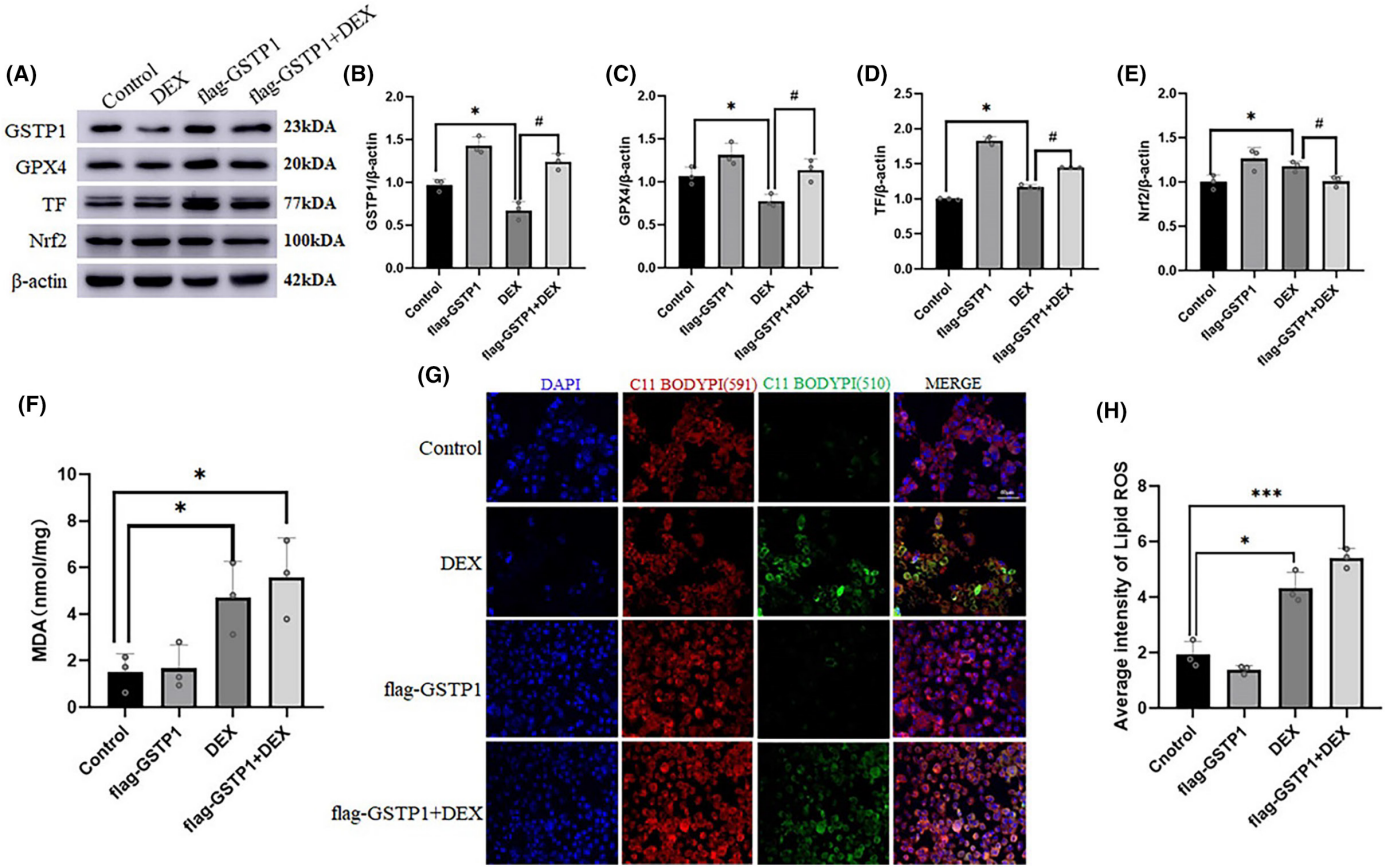
Overexpression of GSTP1 could reverse low expression of GPX4, while not activate Nrf2 or alleviate increasing of TF induced by DEX. (A–E) The expression of Nrf2, GPX4, GSTP1, and TF at the protein level in AML‐12 cells (*n* = 3). (F) The content of MDA in AML‐12 cells of different groups (*n* = 3). (G) and (H) The content of C11 BODIPY in AML‐12 cells of different groups. Scale bar = 60 μm. Values are expressed as the mean ± SEM, *p < 0.05, ***p < 0.001 versus the control group, ^#^ *p* < 0.05 versus the pcDNA‐flag‐GSTP1+DEX group. DEX, dexamethasone.

We further analysed whether GSTP1 overexpression could reduce stress‐induced AML‐12 cell damage. GSTP1 overexpression effectively alleviated the DEX‐induced decrease in GPX4 expression caused by DEX. However, this did not lead to activation or increased expression of Nrf2. Furthermore, it did not inhibit the DEX‐induced increase in TF expression caused by DEX (*p* < 0.05; Figure 6A–E). We also measured lipid ROS and MDA levels in each group and observed that GSTP1 overexpression in AML‐12 cells did not cause oxidative stress damage. However, when GSTP1 was simultaneously overexpressed with DEX, lipid ROS and MDA levels increased (*p* < 0.05) (Figure 6F–H). These data indicate that overexpression of GSTP1 could reverse the low expression of GPX4, without activating Nrf2 or alleviating the increasing in TF. These results indicated that TF/Nrf2/GSTP1 is involved in stress‐induced hepatocellular injury through ferroptosis.

**FIGURE 7:**
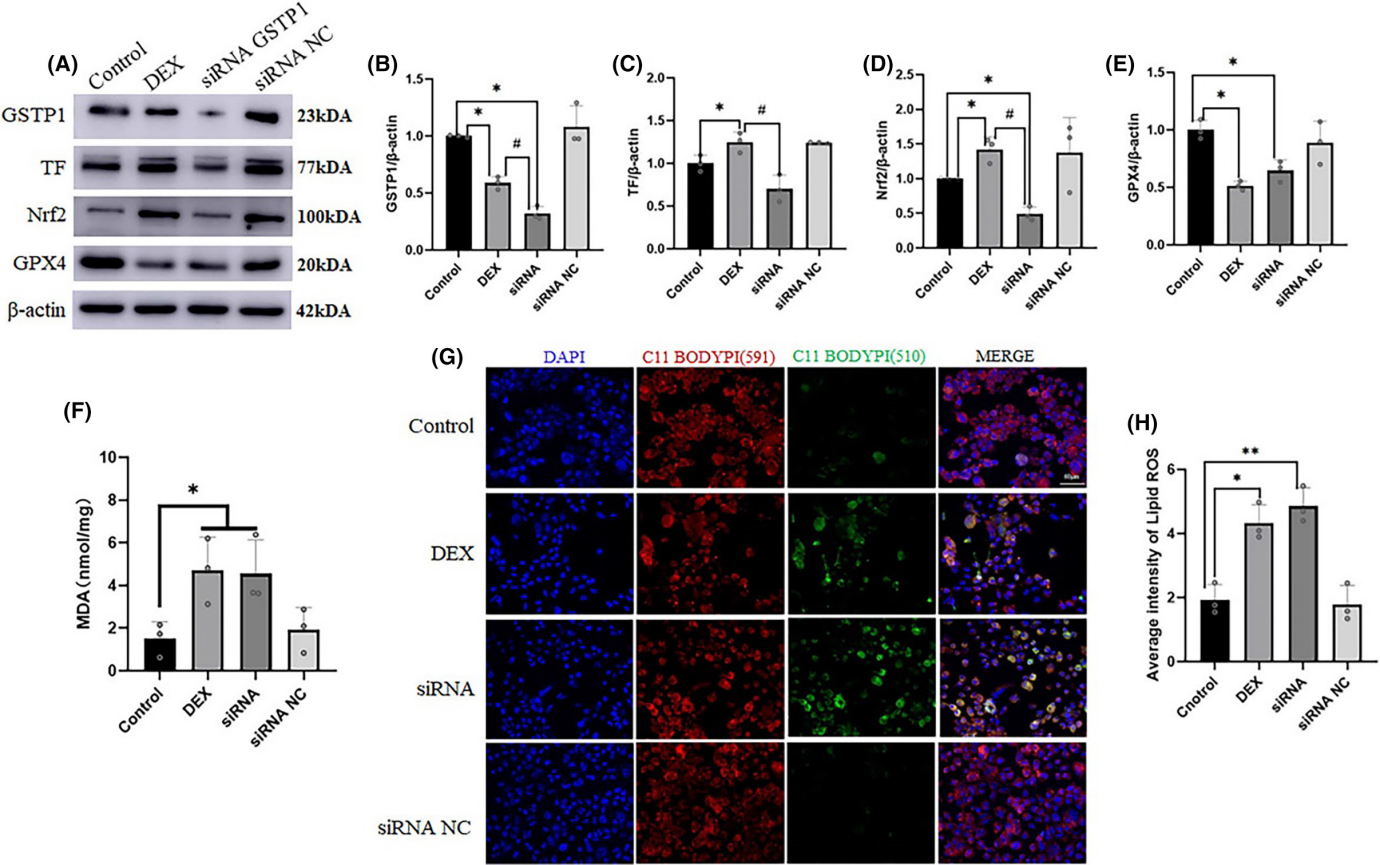
GSTP1 knockdown led to more severe stress damage in AML‐12 cells. (A–E) The expression of Nrf2, GPX4, GSTP1, TF at the protein level in AML‐12 cells (*n* = 3). (F) The content of MDA in AML‐12 cells of different groups (*n* = 3). (G) and (H) The content of C11 BODIPY in AML‐12 cells of different groups. Scale bar = 60 μm. Values are expressed as the mean ± SEM, *p < 0.05, **p < 0.01 versus the control group, ^#^ *p* < 0.05 versus the siRNA group. MDA, malondialdehyde.

To confirm the role of GSTP1 in hepatocyte injury, we used siRNA to knockdown GSTP1 expression in AML‐12 cells. Oxidative stress‐induced damage was more severe in the siRNA group than in the DEX group. In addition, we measured the expression levels of GSTP1, GPX4, TF and Nrf2. We observed a decrease in GSTP1 and GPX4 expression following the siRNA‐mediated GSTP1 knockdown. Notably, TF expression was higher in the DEX group than in the control group but was significantly reduced in the siRNA group. The expression of Nrf2 was higher in DEX group compared with that in the control group; however, following siRNA‐mediated GSTP1 silencing, Nrf2 expression was lower than that in the control group (*p* < 0.05; Figure 7A–E). Furthermore, we measured lipid ROS and MDA levels and found that decreased GSTP1 expression resulted in increased lipid ROS and MDA levels (*p* < 0.05; Figure 7F–H). These results suggested that GSTP1 knockdown led to severe stress damage in hepatocytes.

## 4 DISCUSSION

Elevated GC levels during stress can induce long‐term depression‐like phenotypes in mice, such as anhedonia and despair.^27^, ^28^ Our preliminary research provides evidence that restraint stress leads to notable pathological changes in the central nervous system and affects the regulation of cardiac function in rats.^29^, ^30^, ^31^ However, the effect of stress on hepatocellular injury remains unclear. Elevated levels of AST and ALT in the serum are widely recognized as indicators of liver dysfunction.^32^ In this study, we observed elevated serum ALT and AST levels and reduced hepatic blood flow induced by restraint stress. Additionally, HE staining revealed restraint stress‐induced hepatocellular swelling. These results suggested that restraint stress leads to liver dysfunction and hepatocellular injury.

Metabolomic analysis was conducted to investigate the alterations in hepatic metabolism caused by restraint stress. These results indicate that restraint stress causes metabolic disturbances in the liver, particularly affecting amino acid biosynthesis. Amino acid biosynthesis plays crucial roles in various cellular metabolic processes. Reduced cysteine synthesis leads to decreased glutathione synthesis, which results in GPX4.^33^ It can convert lipid peroxides into non‐toxic lipid alcohols, thereby inhibiting ferroptosis.^34^ Through a comprehensive examination of the KEGG enrichment pathway analysis, it was observed that restraint stress significantly modified L‐y‐glutamylcysteine levels within the GSH metabolic pathway, thereby altering GSH metabolism. Upon exposure to restraint stress, the liver exhibits a significant elevation in T‐GSH, minimal changes in the levels of GSSG, and low expression of GPX4. These findings suggest that stress disrupts the liver metabolism. GSTP plays a crucial role in the transport of electrophilic compounds or reactive aldehydes, facilitating the conversion of reduced GSH to its oxidized form, GSSG.^35^ The expression of GSTP1 is observed in various tissues and is associated with the metabolism of diverse carcinogenic compounds, protection against DNA damage, and activation of tumour suppressor genes.^36^ GSTP1 promotes the binding of GSH to various ROS, hydrogen peroxide, and toxic substances, thereby protecting hepatocytes against oxidative stress.^37^ Following the initial conformational rearrangement of GSH, GSTP1 facilitates proton transfer between the cysteinyl thiol (GSH‐SH) and glutamyl carboxylate (GSH‐COO‐) moieties of GSH.^26^ Increased methylation of GSTP1 results in its inactivation, which promotes the development of liver cancer.^19^ Our findings indicate that restraint stress caused hypermethylation gene and low expression of the *GSTP1*. In conclusion, restraint stress causes an imbalance in GSH redox activity and may be associated with GPX4 and GSTP1.

GSH directly affects oxidative respiratory chain processes within the mitochondria. Using a TEM, we observed that restraint stress induced mitochondrial membrane dissolution, blurring, and accumulation of cytoplasmic lipid droplets. Ferroptosis is triggered by the generation of lipid ROS and MDA.^38^ In the present study, we observed significant increases in lipid ROS and MDA levels following restraint stress. The intracellular iron levels were maintained at a steady state. When exposed to specific stressors, intracellular iron concentrations increase, promoting Fenton reactions within the cells. The excessive accumulation of lipid peroxides (lipid ROS and MDA) leads to ferroptosis.^39^, ^40^, ^41^ We found that intracellular iron levels in the liver significantly increased in the restraint stress group. Nrf2 upregulates the expression GPX4^42^ and activation of the intracellular antioxidant system. However, Shen^43^ found that inflammatory response‐induced ferroptosis was mediated by the downregulation of GPX4 and upregulation of Nrf2. Our study also demonstrated high Nrf2 and low expression in GPX4 subjected to restraint stress. TF bind iron ions and transport them to other tissues.^44^ Restraint stress led to high TF expression in liver tissue. Polyunsaturated fatty acids (PUFAs) are prone to lipid peroxidation and play substantial roles in ferroptosis.^45^ ACSL4 plays a crucial role in the biosynthesis and remodelling of phosphatidylethanolamine (PE), activating PUFAs and influencing their transmembrane properties. When catalysed by fatty acid oxygenases, PUFA‐PE undergoes oxidative reactions, leading to ferroptosis.^46^ Our study found that the expression of ACSL4 decreased after restraint stress. ACSL4 expression is suppressed during the early stages of ischemic stroke, while overexpression of ACSL4 exacerbated ischemic brain injury.^47^ Therefore, we believe that the low ACSL4 expression induced by binding stress occurs in the early stages of liver injury. Combined with the above results, we conclude that elevated iron ions in the liver potentially enter hepatocytes through TF and transferrin receptors, activating Nrf2 and leading to disrupted lipid peroxidation and impaired GSH redox homeostasis through GPX4 and GSTP1.

Fer‐1, a well‐established inhibitor of ferroptosis.^22^ We found that the results treatment with Fer‐1 were similar to those in the control group in various aspects, including the expression of TF, GPX4, ACSL4, GSTP1 and Nrf2, macroscopic observations, HE staining, and the production of lipid ROS. These results suggest that GSTP1 and Nrf2 are involved in ferroptosis induced by restraint stress in hepatocytes, and that GSTP1 may play a protective role.

To further understand the specific mechanisms by which GSTP1 contributes to stress‐induced hepatocellular damage and its relationship with Nrf2, GPX4 and TF. We used DEX induced AML‐12 cells to establish a cellular model of restraint stress. Additionally, Fer‐1 and the Nrf2 inhibitor ML‐385^48^ were included as control interventions. DEX treatment led to the activation of Nrf2 in AML‐12 cells, resulting in decreased GSTP1 expression. Additionally, there was an increasing trend in MDA and lipid ROS levels in the DEX group. Notably, Fer‐1 effectively blocked DEX‐induced changes in the expression of GSTP1, GPX4 and TF caused by DEX. In contrast, ML‐385 only suppressed the upregulation of Nrf2. Although there was no significant difference in TF expression between the control and ML‐385+DEX‐treated groups, the expression of GSTP1 and GPX4 decreased, and the levels of MDA and lipid ROS accumulated in AML‐12 cells. These results provide evidence for the important role of Nrf2 in ferroptosis in DEX‐induced hepatocyte injury. To explore the relationship between Nrf2 and GSTP1, AML‐12 cells were simultaneously exposed to ML‐385, Fer‐1 or DEX. ML‐385 blocked alterations in GSTP1, GPX4 and TF expression, which were otherwise mitigated by Fer‐1. This suggests that Nrf2 activation positively regulates GSTP1 and GPX4 expression. However, owing to the severe hepatocellular damage observed in this study, the activation of Nrf2 and its transcriptional activity in promoting GPX4 and GSTP1 expression could not fully compensate for the overall reduction caused by stress.

To gain a comprehensive understanding of the interactions between GSTP1, Nrf2, and GPX4, we used pcDNA‐flag‐GSTP1 plasmid^30^ to overexpress GSTP1 in AML‐12 cells. We then meticulously examined the expression of GSTP1, Nrf2, TF and GPX4, as well as the production of MDA and lipid ROS. GSTP1 overexpression suppressed the changes in GPX4 and Nrf2 expression induced by DEX, while still leading to an elevation in TF expression. These findings suggest that GSTP1 overexpression does not activate DEX‐induced Nrf2. Antioxidant enzymes, such as SOD1, SOD2, catalase, GPX and GSTP1 can effectively reduce ROS accumulation in cells.^49^ Notably, despite the inhibitory effect of GSTP1 overexpression on changes in the expression of ferroptosis‐related proteins induced by DEX, the accumulation of MDA and lipid ROS did not return to normal levels in the control group. However, GSTP1 is not the sole line of defence against ROS degradation,^50^ it is possible that GSTP1 overexpression only partially inhibits the expression of ferroptosis‐related proteins induced by DEX, while failing to completely mitigate lipid peroxidation triggered by DEX. In this study, we further analysed the role of GSTP1 in AML‐12 cells using siRNA to knockdown its expression. Our results revealed that downregulation of GSTP1 led to more severe stress damage than in the DEX group. These findings suggest that GSTP1 plays an important role in stress injury in AML‐12 cells and is involved in the downstream physiological functions of TF and Nrf2. Specifically, TF is responsible for the transport of Fe^3+^ into cells, leading to the activation of Nrf2 and the subsequent induction of GSTP1 expression, thus contributing to the antioxidative system within cells. Notably, decreased GSTP1 expression hindered the expression and functional roles of TF and Nrf2. Furthermore, examination of MDA and lipid ROS levels in AML‐12 cells following GSTP1 interference demonstrated notable accumulation, surpassing the levels in the DEX group. This observation further supported the antioxidant function of GSTP1 in ferroptosis.

**FIGURE 8:**
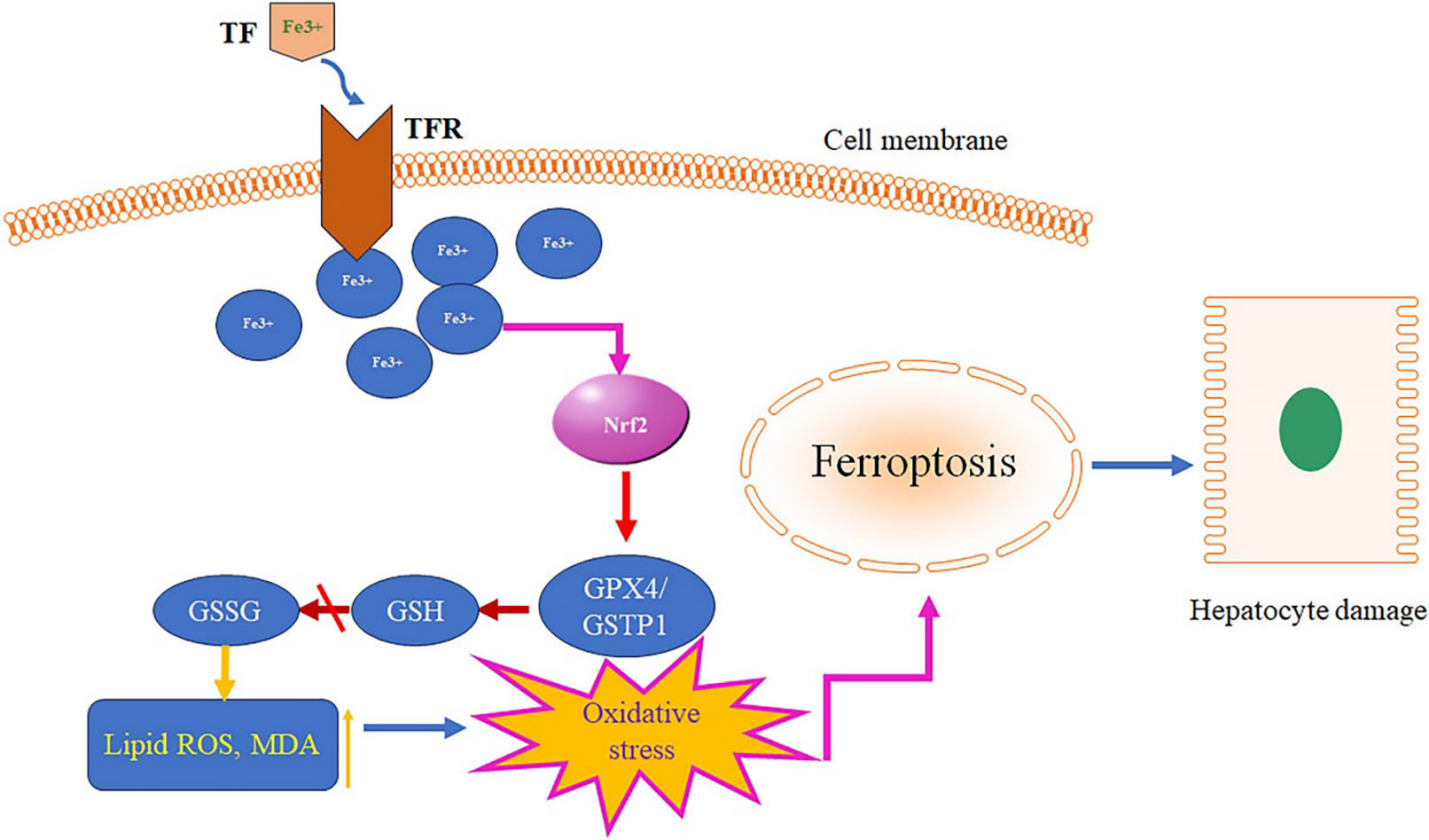
GSTP1 involvement in the stress‐induced hepatocyte ferroptosis process via TF/Nrf2.

In summary, our findings suggest that restraint stress induces a significant influx of iron ions into hepatocytes through TF and its receptors and activates Nrf2. This activation of Nrf2 subsequently affected the expression of GPX4 and GSTP1. Consequently, the conversion of reduced GSH to GSSG was inhibited, disrupting the redox equilibrium of GSH. This disruption triggered the aggregation of lipid ROS and MDA, ultimately leading to ferroptosis and subsequent hepatocellular injury (Figure 8).

## 5 CONCLUSION

This study revealed that restraint stress causes hepatic dysfunction and morphological abnormalities in mice. This is accompanied by alterations in markers related to ferroptosis as well as increased methylation and reduced expression of GSTP1. Furthermore, restraint stress led to a reduction in the expression of GPX4, while Nrf2 expression was elevated. Notably, the addition of an Nrf2 inhibitor suppressed the expression of GSTP1, GPX4 and TF, whereas GSTP1 overexpression inhibited the stress‐induced increase in Nrf2 expression. The results of this study suggest that the TF/Nrf2/GSTP1 pathway may be a significant mechanism underlying the development of pathological liver damage induced by restraint stress.

## AUTHOR CONTRIBUTIONS

**Xiaofei Tian:** Formal analysis (equal); investigation (equal); validation (equal); visualization (equal); writing – original draft (equal). **Yingmin Li:** Methodology (equal); supervision (equal). **Lei Lei:** Formal analysis (equal); visualization (equal). **Xiaowei Feng:** Visualization (equal). **Hongjian Xin:** Visualization (equal). **Hao Chen:** Software (equal). **Guozhong Zhang:** Resources (equal). **Min Zuo:** Supervision (equal). **Weibo Shi:** Funding acquisition (equal); supervision (equal); writing – review and editing (equal). **Bin Cong:** Funding acquisition (equal); resources (equal); supervision (equal); writing – review and editing (equal).

## FUNDING INFORMATION

This work was supported with funds from the Key Program of National Natural Science Foundation of China (82130055), the Major Program of National Natural Science Foundation of China (82293651) and the National Natural Science Foundation of China (82072109).

## CONFLICT OF INTEREST STATEMENT

The authors declare no conflict of interest.

## INSTITUTIONAL REVIEW BOARD STATEMENT

All procedures were conducted in accordance with the National Institutes of Health guidelines and were approved by the Institutional Review Board for Animal Experiments at Hebei Medical University (IACUC‐Hebmu‐2023011).

## CONSENT

Not applicable.

## Data Availability

The data that support the findings of this study are available from the corresponding author upon reasonable request.
